# Evolution of Physical Status From Diagnosis to the End of First-Line Treatment in Breast, Lung, and Colorectal Cancer Patients: The PROTECT-01 Cohort Study Protocol

**DOI:** 10.3389/fonc.2020.01304

**Published:** 2020-08-07

**Authors:** Joris Mallard, Elyse Hucteau, Roland Schott, Thierry Petit, Martin Demarchi, Christine Belletier, Meher Ben Abdelghani, Hélène Carinato, Pascale Chiappa, Cathie Fischbach, Michal Kalish-Weindling, Audren Bousinière, Stéphane Dufour, Fabrice Favret, Xavier Pivot, Thomas J. Hureau, Allan F. Pagano

**Affiliations:** ^1^Institut de Cancérologie Strasbourg Europe (ICANS), Strasbourg, France; ^2^EA 3072: Mitochondria, Oxidative Stress and Muscular Protection Laboratory, Faculty of Medicine, Faculty of Sports Sciences, University of Strasbourg, Strasbourg, France

**Keywords:** cachexia-fatigue vicious cycle, cachexia, cancer-related fatigue, exercise intolerance, muscle wasting, tumor

## Abstract

**Background:** Cancer cachexia and exacerbated fatigue represent two hallmarks in cancer patients, negatively impacting their exercise tolerance and ultimately their quality of life. However, the characterization of patients' physical status and exercise tolerance and, most importantly, their evolution throughout cancer treatment may represent the first step in efficiently counteracting their development with prescribed and tailored exercise training. In this context, the aim of the PROTECT-01 study will be to investigate the evolution of physical status, from diagnosis to the end of first-line treatment, of patients with one of the three most common cancers (i.e., lung, breast, and colorectal).

**Methods:** The PROTECT-01 cohort study will include 300 patients equally divided between lung, breast and colorectal cancer. Patients will perform a series of assessments at three visits throughout the treatment: (1) between the date of diagnosis and the start of treatment, (2) 8 weeks after the start of treatment, and (3) after the completion of first-line treatment or at the 6-months mark, whichever occurs first. For each of the three visits, subjective and objective fatigue, maximal voluntary force, body composition, cachexia, physical activity level, quality of life, respiratory function, overall physical performance, and exercise tolerance will be assessed.

**Discussion:** The present study is aimed at identifying the nature and severity of maladaptation related to exercise intolerance in the three most common cancers. Therefore, our results should contribute to the delineation of the needs of each group of patients and to the determination of the most valuable exercise interventions in order to counteract these maladaptations. This descriptive and comprehensive approach is a prerequisite in order to elaborate, through future interventional research projects, tailored exercise strategies to counteract specific symptoms that are potentially cancer type-dependent and, *in fine*, to improve the health and quality of life of cancer patients. Moreover, our concomitant focus on fatigue and cachexia will provide insightful information about two factors that may have substantial interaction but require further investigation.

**Trial registration:** This prospective study has been registered at ClinicalTrials.gov (NCT03956641), May, 2019.

## Introduction

Hallmark symptoms in patients with cancer include exacerbated fatigue (1, 2) and muscle wasting (3, 4) which impair their quality of life (2, 5, 6). Importantly, these are due to both the disease and the antineoplastic treatments (2, 4, 6–8), resulting in substantial differences across cancer types (3, 8–12). The characterization of these key parameters and, most importantly, their evolution throughout cancer treatment may represent the first step in finding ways of efficiently counteracting their development.

Cancer-related fatigue (CRF) is considered the main symptom in cancer patients and can be defined by clinicians as a chronic sensation of tiredness that is not fully reversed by rest, unlike common fatigue experienced by healthy individuals (1, 2), and is assessed subjectively with questionnaires. Moreover, as a multi-factorial symptom, CRF is also documented by objective measurements of neuromuscular fatigue, such as changes in force output (13). Previous studies report that 60–96% of cancer patients experience CRF (14), a symptom that persists even after treatments conclude (8, 15). As a consequence, daily activities are affected (16) and physical activity levels are largely decreased (17, 18), affecting, in turn, skeletal muscle mass and function.

**Figure 1:**
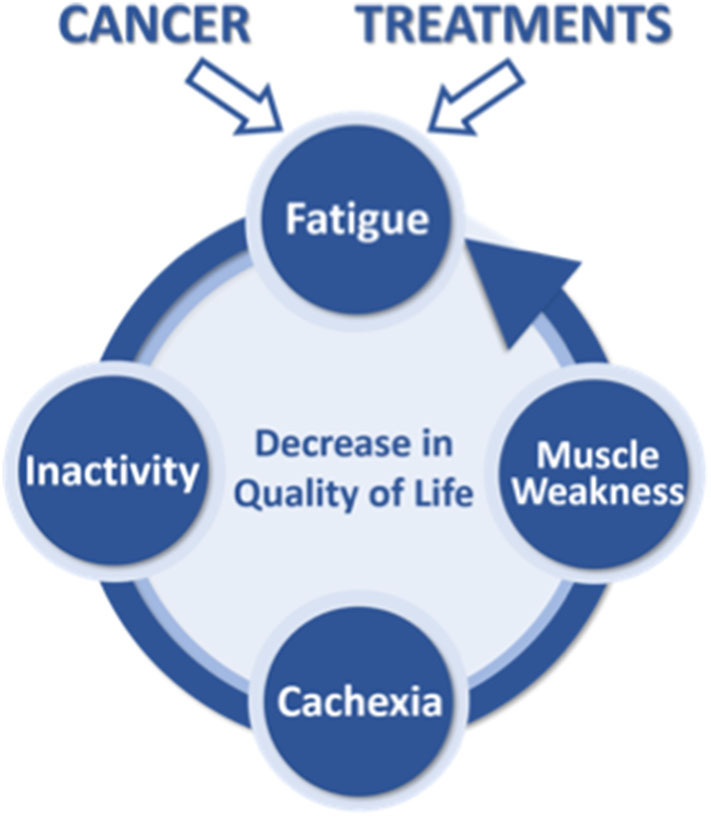
Illustration of the theoretical cachexia–fatigue vicious cycle.

Skeletal muscle plays a major function in daily activity and possesses the ability to adapt according to applied stresses. This characteristic plasticity expresses itself through both structural and metabolic adaptations, and it is well-known that a decrease in muscle activity will lead to muscle deconditioning (19–23). In the context of cancer patients, the combination of a decrease in muscle mass and strength, an increase in fatigue, and a perturbation of identified biomarkers (i.e., inflammation, albumin, and hemoglobin) is known as cachexia (3, 24–26). It is a multifactorial syndrome characterized by ongoing loss of skeletal muscle mass (with or without loss of fat mass) that cannot be fully reversed by conventional nutritional support, leading to progressive functional impairment (4). Cachexia is considered a major public health problem and another important symptom in cancer patients (27). Indeed, a high level of cachexia is known to be associated with poor quality of life (6), reduced survivability (28), and massive public health costs (29). Cachexia also negatively impacts both perceived and/or objective CRF (5, 30–32), thus placing cancer patients in the center of a vicious cycle with fatigue inducing cachexia and cachexia inducing fatigue (Figure 1). This cachexia–fatigue vicious cycle suggests a strong interconnection between CRF and cachexia.

Exercise interventions have been investigated for improving CRF in many studies (33), with positive effects and a widespread agreement that they represent some of the most efficient interventions in cancer patients (34, 35). Exercise interventions also appear to be the best interventions to counteract cachexia specifically (36–38). For example, several studies have shown that exercise interventions in cancer patients are able to increase anti-inflammatory cytokine expression (IL-10, IL-15) and therefore decrease systemic pro-inflammatory factors (TNF-α, IL-6) responsible, at least in part, for the unbalanced protein turnover signaling and muscle deconditioning observed in this population (36–38). Today, exercise interventions are increasingly being recognized as an efficient strategy in counteracting both CRF and cachexia in cancer patients. Indeed, the implementation of supportive care with adapted physical activity in cancer patients is part of current and future political recommendations in France (39, 40) and elsewhere (41–43).

However, despite a great number of studies showing the positive effects of exercise interventions on many different health parameters (44–46); the literature also highlights the significant variability of patient adaptation to exercise programs (33, 47, 48). This variability appears to be accountable for the lack of tailored exercise interventions (49) and occurs due to the large number of parameters that influence the effects of exercise prescribed to patients: the type and stage of cancer, the type of treatment, and the physical status at the time of diagnosis. For example, lung cancer patients present a higher risk of cachexia, whereas breast cancer patients present a higher risk of heart failure (9, 50). All these different parameters, and their evolution throughout treatment, should be considered in prescribing the best exercise strategies on an individual basis, as opposed to generic exercise programs.

In this context, the aim of our study will be to describe, in cancer patients, the evolution of their physical status from diagnosis until the end of first-line treatment. This prospective cohort study will include patients with lung, colorectal, and breast cancer as they represent ~30% of all newly diagnosed cancers (51).

## Materials and Methods

### Study Population

As they represent ~30% of all newly diagnosed cancers (51), this cohort study will focus on three different cancer types: breast, lung, and colorectal. The detailed inclusion and exclusion criteria are summarized in Table 1.

**Table 1:** Inclusion and exclusion criteria.

**Inclusion criteria**	**Exclusion criteria**
• First-line cancer treatment	• Psychiatric, musculoskeletal, or neurological disorders
• Breast cancer, Stage II or III, treated by taxane-based CT	• Pregnant or nursing women
• Colorectal cancer, stage IV, treated by CT, RT, IT, or TT	
• Non-small cell lung cancer, stage III or IV, treated by CT, RT, IT, or TT	
• Age ≥18 years	
• Performance status WHO 0–2	
• Life expectancy >6 months	
• Ability to speak, understand and read French	
• No contraindication to physical assessments	
• Affiliate to social security system	
• Give written inform consent	

*CT, chemotherapy; IT, immunotherapy; RT, radiation therapy; TT, targeted therapy; WHO, World Health Organization performance status*.

Patients newly diagnosed with cancer by an oncologist and satisfying inclusion and exclusion criteria described in Table 1 will be informed of the study protocol. If interested, the patient will contact one of the study coordinators for full information about the study as well as to discuss any questions pertaining to it. Once the patient has provided the written consent form to participate in this study, the visits will be scheduled.

### Study Design

**Figure 2:**
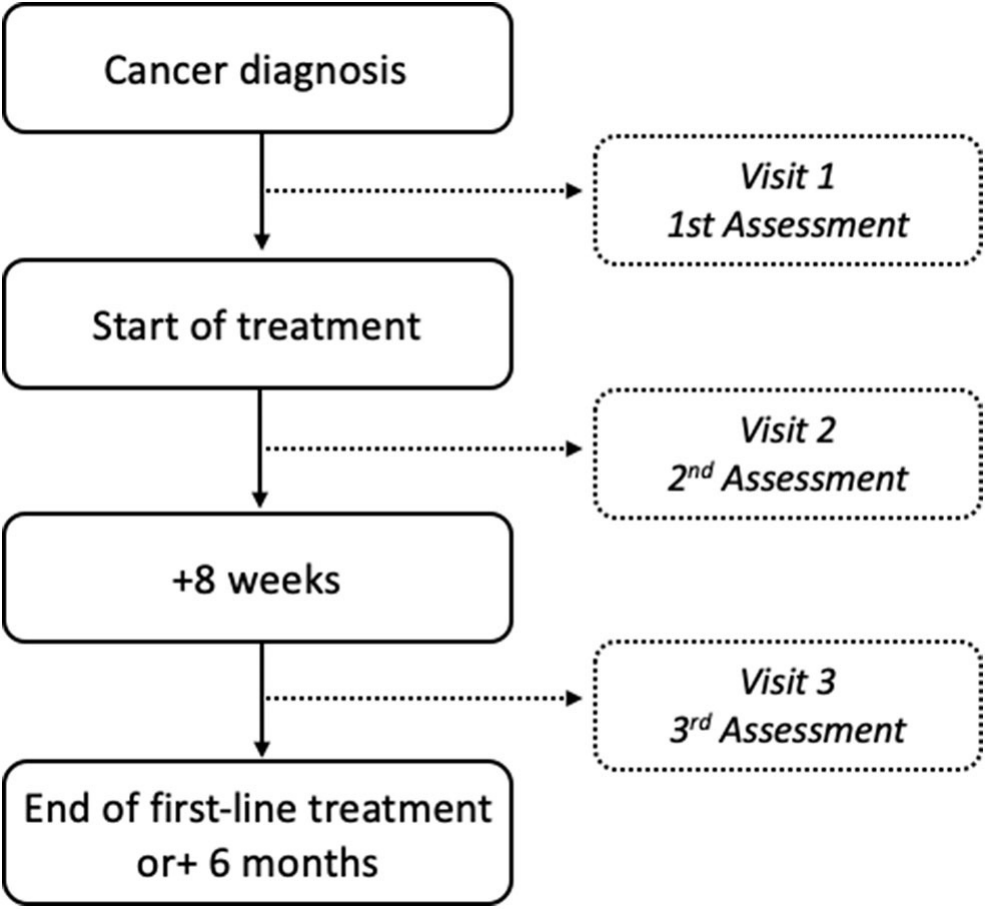
Flowchart of the PROTECT-01 study design.

This cohort study aims to investigate the evolution of physical status from diagnosis to the end of first-line treatment. Therefore, after signing the written consent form, three visits will be scheduled. All assessments, detailed hereafter, will be performed at inclusion (Visit 1, between the time of diagnosis and the beginning of the treatment), 8 weeks after the beginning of the treatment (Visit 2), and either during the week after completion of first-line treatment or at the 6-months mark (Visit 3), whichever occurs first. The PROTECT-01 enrolment time is 18 months, and the study design is detailed in Figure 2.

### Study Assessments

In order to assess the evolution of our different measurements throughout cancer treatment, all assessments will be performed via the same methods and under the same conditions across the three visits. All of the assessments will be performed chronologically in the order that they appear in the description provided hereafter. All parameters analyzed in this study are displayed in Table 2.

**Table 2:** Study time points.

	**Before the start of treatment**	**8 weeks after the start of treatment**	**End of treatment or 6 months**
	**Visit 1** **1st assessment**	**Visit 2** **2nd assessment**	**Visit 3** **3rd assessment**
Inclusion criteria check-up	X		
Informed consent	X		
**Clinical data**			
Participant information	X		
Disease history	X		
Cancer record	X	X	X
Cancer treatment	X	X	X
Performance status WHO	X	X	X
Concurrent treatments	X	X	X
**Questionnaires**			
FACIT-F	X	X	X
GPAQ	X	X	X
EORTC QLQ-C30	X	X	X
**Physical assessments**			
Body composition	X	X	X
Cachexia		X	X
Respiratory function	X	X	X
Maximal voluntary force	X	X	X
Fatigability	X	X	X
Physical performance	X	X	X
Exercise tolerance	X	X	X

*Visit 1, between the time of diagnosis and the beginning of the treatment; Visit 2, 8 weeks after the beginning of the treatment; Visit 3, at the end of first-line treatment or at the 6-months mark; EORTC QLQ-C30, European Organization for Research and Treatment of Cancer Quality of Life Core Questionnaire; FACIT-F, Functional Assessment of Chronic Illness Therapy–Fatigue; GPAQ, Global Physical Activity Questionnaire; WHO, World Health Organization performance status*.

### Clinical Data

Age, gender, tobacco consumption, significant health history (e.g., disease, surgery), type of cancer, and date of diagnosis will be collected via the medical file at the first visit. The presence of metastases and their sites, type of treatment (chemotherapy, immunotherapy, targeted therapy, radiotherapy) for lung and colorectal cancer patients, disease stage, performance status WHO, and significant concomitant treatments (e.g., corticosteroids, analgesics, antidepressants, antihypertensives) will also be collected and tracked throughout the study.

### Questionnaires

Perceived cancer-related fatigue will be reflected by the score of the Functional Assessment of Chronic Illness Therapy–Fatigue (FACIT-F) scale version 4 (52). The FACIT-F is a subscale of the FACIT scales, a collection of health-related quality of life questionnaires targeted to the management of chronic illness. Patients will answer a 13-item scale to report their fatigue and its impact on their daily life during the previous week. Items are rated on a 0–4 intensity scale (0: not at all, 1: a little bit, 2: somewhat, 3: quite a bit, 4: very much). After performing reversal scores for appropriate items, scores will be analyzed through two specific subscales (health-related quality of life and fatigue) and the global score obtained from the FACIT-F, where higher scores indicate less fatigue or better functioning.

Physical activity level will be assessed using the 16-item Global Physical Activity Questionnaire version 2 (GPAQ-2) (53). GPAQ-2 collects information on sedentary behavior and physical activity in three domains: (1) activity at work, (2) travel to and from places, and (3) recreational activities during a typical week. Patient engagement in moderate and vigorous physical activity level is assessed based on dichotomous response (i.e., “yes” or “no”). Intensity of activities are classified using MET (Metabolic Equivalent of Task) as follows: inactivity (1 MET), moderate (4 METs), and vigorous (8 METs). For patients performing moderate and/or vigorous activities, they will also have to report their frequency (number of days per week) and duration. Then, a score will be calculated based on the characteristics (i.e., MET, frequency, and duration) of activities in the moderate and vigorous domains. The higher the score, the better the patient's physical activity level.

Quality of life will be evaluated by the European Organization for Research and Treatment of Cancer (EORTC) Quality of Life Core Questionnaire (EORTC QLQ-C30), version 3, with 30 items (54). The self-administered EORTC QLQ-C30 is specifically designed for cancer patients, and version 3 is intended for all patients with cancer regardless of tumor site. The questionnaire includes five functioning scales (physical, role, cognitive, emotional, and social functioning) and three symptom scales (fatigue, pain, and nausea/vomiting). Additional cancer symptoms (dyspnea, sleep disturbance, loss of appetite, diarrhea, and constipation) and financial difficulties due to cancer and treatments are also reported by separate items. Patients will score intensity of symptoms during the past week for each item on a scale of 1–4 (1: not at all, 2: a little, 3: quite a bit, 4: very much). Patient overall global health and quality of life will be assessed, ranging from 1 to 7 (1 for very poor to 7 for excellent). A total score between 0 and 100 will be calculated for each scale. For global and functioning scales, a higher score is considered better, while for symptom scales a lower score is better.

### Body Composition and Cachexia

Body mass and composition analyses will be performed using a bioelectrical impedance meter (SECA mBCA 515, SECA, Hamburg, Germany), validated in comparison to different robust methods assessing body composition, such as air-displacement plethysmography, dual-energy X-ray absorptiometry, and deuterium dilution (55). The bioelectrical impedance meter is also regularly used in studies investigating body composition in cancer patients (56–59). We will quantify body mass, fat free mass, fat mass, skeletal muscle mass, visceral fat, and body water repartition. Patient height will be measured to calculate body mass index (BMI, body mass/height^2^).

In accordance with Fearon et al. (4), cachexia will be diagnosed using fat-free mass index cut-off (i.e., <14.6 kg/m^2^ for men and <11.4 kg/m^2^ for women) assessed by a bioelectrical impedance meter (4). Furthermore, the severity of the cancer-related body mass loss (i.e., cachexia) will be investigated using the Martin score (28) at the second and third visit. Martin score is a robust grading system incorporating the independent prognostic significance of both BMI and percentage of body mass loss based on a correlation matrix from a cohort of more than 8,000 cancer patients (28). A severity stage of body mass loss between 0 and 4 is attributed to each patient according to their BMI at cancer diagnosis (28), with stage 0 being the lowest severity stage of cachexia, and stage 4, the highest. Importantly, each score is associated with a specific cumulative survival curve: the lower the score, the greater the survival probability (28).

### Respiratory Function

Respiratory function will be assessed using a portable spirometer (USB Electronique Portable *Spirobank II* Smart MIR, Rome, Italy). Patients will have to take maximum inspiration and blow to maximum exhalation. The mouthpiece on the device will be changed between each patient. The measured parameters will be: forced expiratory volume in the first second (FEV1), forced vital capacity (FVC), Tiffeneau index or FEV1% (FEV1/FVC), peak exploratory flow (PEF), mean forced expiratory flow between 25 and 75% of FVC (FEF 25–75%), inspiratory vital capacity (IVC), expiratory vital capacity (EVC), inspiratory capacity (IC), expiratory reserve volume (ERV), and tidal volume (TV).

### Maximal Voluntary Force

**Figure 3:**
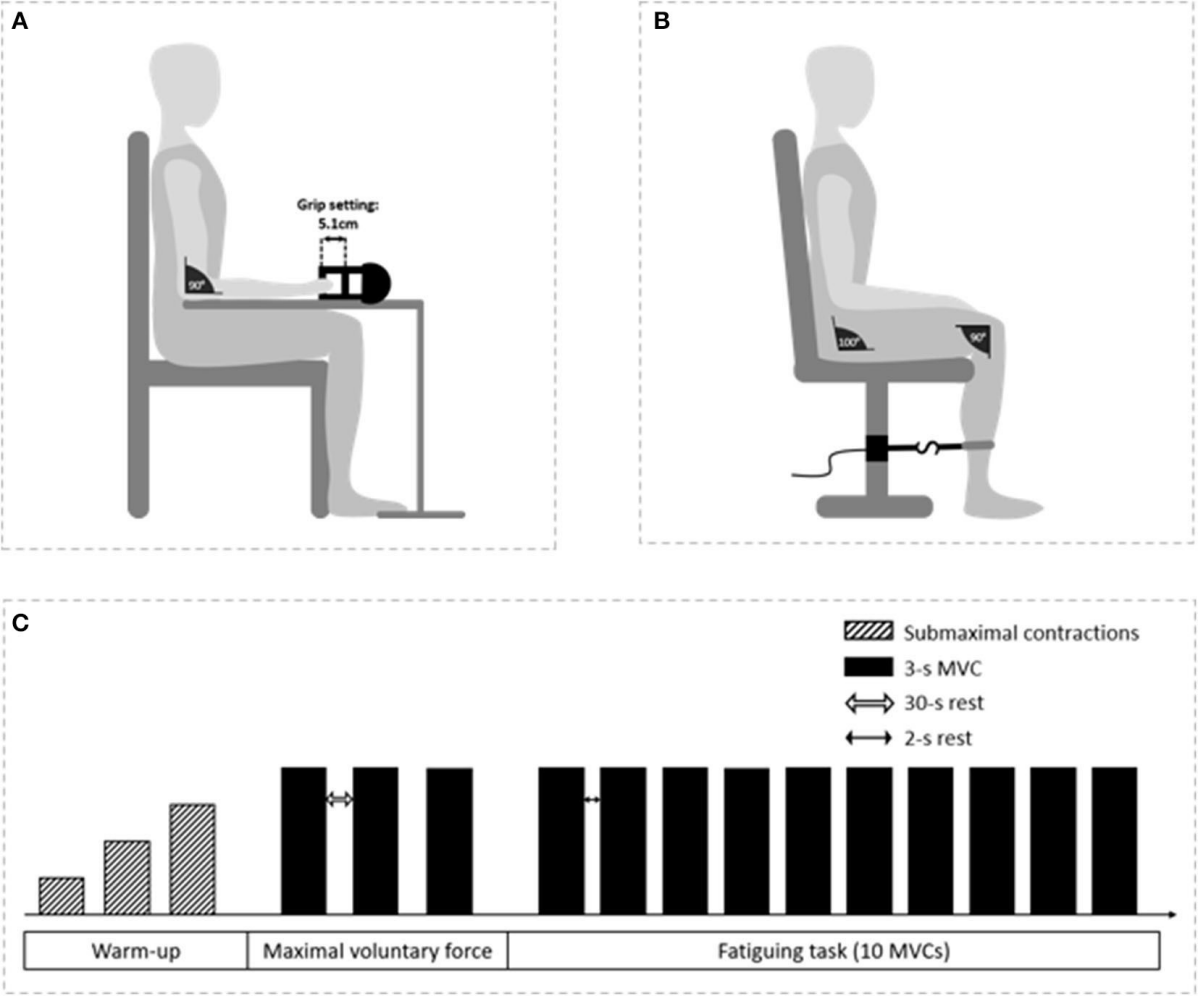
Illustration of the neuromuscular assessments. Participant's position for finger flexor **(A)** and knee extensor **(B)** assessments and schematic representation of the protocol **(C)**.

Finger flexors force will be measured using a handgrip dynamometer (Takeï, TK200, Takei Scientific Instruments, Tokyo, Japan). Patients will sit upright in a chair with their feet touching the ground. The dominant arm will be placed with the elbow flexed at 90° on a table, while the forearm and the hand will be placed in a neutral position (Figure 3A). The non-dominant arm will be relaxed in a neutral position. For standardization, grip settings will be set at 5.1 cm (Figure 3A), corresponding to the standard position for grip testing (60, 61). Knee extensors force will be measured with a force transducer (Force sensor kit, Chronojump, Barcelona, Spain) positioned on a leg extension device (ProForma—Bodytone Evolution Extensions, Barcelona, Spain) (Figure 3B). Patients will be assessed in a seated position with the hip and knee joints fixed at 100 and 90°, respectively (where 180° represents a full knee extension), and aligned in the frontal axis. The lower leg will be attached with a non-compliant strap right above the malleoli. All participants will be familiarized with the maximal voluntary isometric contractions until the observation of a force plateau, indicating the ability to maintain the maximal contraction over 3 s and consistent peak values (≤5% difference) between trials. After a standardized warm-up (three contractions at 25, 50, and 75% of the estimated maximal voluntary contraction [MVC] torque), patients will be asked to perform three 3-s maximal voluntary isometric contractions under verbal encouragement. A 30-s rest period will be given between each MVC. The maximal value measured during the three trials will be used for further analysis.

### Fatigability

Patients will be placed in the same position as the maximal voluntary force assessment. For both finger flexors and knee extensors, fatigability will be assessed by performing ten 3-s maximal voluntary isometric contractions under verbal encouragement with a 2-s rest period between each contraction. Fatigability will be calculated as the percent difference in peak force between the first and the last contraction (62).

### Physical Performance

Physical performance will be assessed using the Short Physical Performance Battery (SPPB), a validated and reproducible test (interclass correlation coefficient of 0.86), indicating the risk of skeletal muscle force and mass loss in older adults (i.e., sarcopenia) (19, 63, 64). The SPPB test includes balance tests (joint feet, semi-tandem feet, and tandem feet), a 4-m walking speed test, and a 5-times-sit-to-stand test. For the balance tests, patients will be timed over a 10-s period and stopped if they lose their balance during this time. For the walking speed test, patients will have to walk spontaneously over 4 m, two times, and the fastest trial, in m·s^−1^, will be used for further analysis. For the 5-times-sit-to-stand test, a chair (height, 43–45 cm; depth, 47.5 cm) will be used. With their arms crossed over their chest, patients will have to sit and stand five times as fast as possible on two different trials. Here again, the best performance will be used for analysis.

### Exercise Tolerance

Exercise tolerance will be assessed using the classical 6-min walking test (6-MWT), following the American Thoracic Society (ATS) Statement recommendations (65). The 6-MWT is a validated and reproducible test (interclass correlation coefficient of 0.85), associated with mortality in cancer patients (66, 67). Patients will walk in a covered, flat, straight, well-delineated (marked every 3 m) and with no disturbance, 20-m corridor. A starting line will be marked, and two cones will represent the location of the U-turns. Physiological measurements (mean arterial pressure, oxygen saturation, and heart rate) will be performed at rest, at least 10 min before the beginning of the test, and again immediately at the end of the 6-MWT. Instructions will systematically be given to the patient as recommended in the ATS Statement (65). The total distance covered during the test (in meters) will be used to assess exercise tolerance.

### Sample Size

An *a priori* sample size estimation was performed using the following sample size calculation formula (68):

$$\begin{array}{l} Sample\;size\;=\;\frac{{Z_{-a/2}}^{2}SD^{2}}{d^{2}} \end{array}$$

Where: *Z*_−*a*/2_ is a standard normal variate (at 5% type 1 error i.e., *P* < 0.05, it is 1.96); *SD* is the standard deviation of the main variable based on previous study; and *d* is the absolute error or precision based on previous study.

The primary outcome of this study is to investigate the evolution of exercise tolerance using the 6-MWT distance. According to Granger et al. (69), a 9.5% decline in the walking distance (i.e., exercise performance) during the 6-MWT test is sufficient to show a clinical decrease in exercise tolerance. Thus, based on the evolution of 6-MWT distances between the first and the second visit in our study, the hypothesis was that cancer patients will reduce, by at least 9.5%, their 6-MWT performance, and thus exhibit signs of exercise intolerance. We also determined the expected baseline 6-MWT results (659 ± 62 m) from the results observed in a healthy subjects group aged 55–75 years old (70), and a 7-m precision was calculated according to a test-retest reliability performed by Demers et al. (67). Using an α level of 0.05 and a power (1-β) of 0.95, the total sample size was calculated as follows:

$$\begin{array}{l} Sample\;size=\frac{1.96^{2}{\left(62\right)}^{2}}{7^{2}}=301 \end{array}$$

Our sample (*n* = 301) will be equally divided into our three groups of interest: 100 lung cancer patients, 100 breast cancer patients, and 100 colorectal cancer patients.

### Statistical Analysis

Normality and variance homogeneity will be investigated on all collected data using the Shapiro-Wilk test and the Levene test, respectively. Appropriate parametric or non-parametric analyses will then be performed. Statistical significance will be set at *P* < 0.05. All values will be expressed as mean ± standard deviation or median ± range.

A multivariate analysis of variance (MANOVA) will be used on our primary outcome (i.e., exercise tolerance) and secondary outcomes (i.e., quality of life, fatigue, physical activity level, strength, fatigability, body composition, cachexia, physical performance, and respiratory function) to protect against Type I errors arising from multiple comparisons. A two-way mixed-design ANOVA will be used (group [lung cancer – colorectal cancer – breast cancer] × time [first visit – second visit – third visit]). Finally, if a significant difference is found, a multiple-comparison analysis will be performed using an appropriate *post-hoc* test.

Finally, interaction between specific variables will be tested with Pearson's correlation coefficients (*r*^2^). For example, we plan to test the possible correlation between subjective and objective fatigue, or between exercise tolerance/quality of life and body composition.

### Ethics and Dissemination

This prospective three-armed cohort study has been approved by the national ethics committee (2019-A00848-49). All subjects gave written informed consent in accordance with the Declaration of Helsinki. Any amendment to the protocol will require the formal modification and approval by the same local ethics committee that approved the study prior to implementation and will be described transparently in subsequent reports. This study is also registered at ClincalTrials.gov (NCT03956641, first posted in May 2019). Patient recruitment and data collection began in September 2019 and are being conducted at Paul Strauss Center Strasbourg and Cancer Institute Strasbourg Europe, Strasbourg, France.

## Discussion

The aim of the PROTECT-01 study will be to investigate the evolution of the physical status, from diagnosis to the end of first-line treatment, of patients with one of the three most common cancers, namely lung, breast, and colorectal (51). Therefore, the present investigation will identify the nature and severity of maladaptation related to exercise intolerance in these three types of cancer. Our results should contribute to identifying the needs of each group of patients and to determining the most valuable exercise interventions to prescribe (i.e., modality, intensity, etc.) in order to counteract major disease-related physical and physiological alterations.

### Rationale and Novelty

Although the consequences of the disease and the associated treatments on physical and physiological function are not completely unknown, the present study protocol is original in several aspects. First, this study is not dedicated to one specific physiological function or organ but is instead focused on assessments of respiratory and muscular functions, cachexia, muscle fatigue, exercise tolerance, perceived fatigue, and quality of life, in order to better prioritize what to target in future exercise prescriptions (e.g., resistance training). Importantly, the relationship between objective (i.e., neuromuscular) and subjective (i.e., perceived) measures of fatigue is insightful (71, 72) and has been clearly identified as a priority (73). Moreover, objective measurements of fatigue are requisite to investigate mechanistically cancer-related fatigue, while the majority of CRF research is, to date, questionnaire-based (2, 74–78). Indeed, while CRF is the major symptom of the disease, which can last despite clinical remission (15), muscle fatigue has not been as extensively investigated in cancer as it has been in other diseases associated with chronic fatigue, such as multiple sclerosis (62, 79) or heart failure (80).

Another strength of the “global” approach of our study is the concomitant interest in CRF and cachexia, two major symptoms (81) that may have substantial interaction (5, 30–32), as suggested in the vicious cycle presented in Figure 1. CRF has been shown to promote inactivity (17, 18), leading to muscle deconditioning (19–23) mainly characterized by a loss in muscle force and mass (4). These rapid muscle alterations are in turn increasing fatigue and fueling this vicious cycle (31). The potential link between these two symptoms seems strong but requires further investigation (72). The PROTECT-01 cohort study is designed to investigate this relationship between CRF and cancer cachexia in cancer patients using repeated measures from diagnosis to the end of first-line treatment. Moreover, the combination of iso-time and iso-status measurements between the three cancers investigated (i.e., after 8 weeks of treatment and after the first-line treatment, respectively) will be important in comparing the specific maladaptation associated with the type of cancer investigated despite differences in treatment durations. The iso-time comparison will be performed to identify the rate of change for each physical and physiological parameter assessed, while the iso-status comparison will be performed to compare the consequences of first-line treatment on our series of assessments across the three cancers investigated.

### Expected Impact and Perspectives

The PROTECT-01 study is expected to bring to light the nature and severity of maladaptation related to exercise intolerance in the three most common cancers. This descriptive and comprehensive study is requisite in order to design interventional research projects with exercise interventions to target a major symptom or maladaptation observed. Importantly, if “exercise is medicine” (82), it is essential to note that it works, to make an analogy, like a pill. Exercise needs to be prescribed with the appropriate “active principle” (i.e., exercise modality) and “dose” (i.e., exercise intensity and duration) to be effective (83). For example, the prevalence of cachexia is high (~50%) and greater in lung or colorectal cancers compared to breast cancer (3). Therefore, resistance training may be of particular interest for lung or colorectal cancer patients, while breast cancer patients may get more health benefits from aerobic exercise to focus on cardiovascular outcomes (9, 50). However, it should be emphasized that this speculation is done without a global and comparative approach, which is needed for such a conclusion. The goal of the PROTECT-01 cohort study will be to provide this approach and initiate future randomized control trials with specific exercise interventions for various cancer patients according to their circumstances. In a context where the suggestion of a unique exercise modality (i.e., resistance, aerobic, or combined training) as the best strategy for all cancer patients may not be convincing (84), we and others (49, 73, 83) believe that tailored exercise interventions are necessary to optimize cancer patient health outcomes and substantially improve their quality of life.

### Methodological Considerations

In order to assess the evolution of the physical status of the patients, this study will require multiple visits from diagnosis to the end of first-line treatment. Thus, at the first visit, newly diagnosed cancer patients will be tested before starting any treatment, indicating that we will be able to assess effects of the disease at this time. However, subsequent visits will be performed during and at the end of first-line treatment, indicating that our measurements will assess the consequences of both the disease and the associated side effects of the treatment (18, 85, 86). Therefore, it will not be possible to isolate the effects of the disease vs. the treatments on our measures in this study. Regardless, it is more important to characterize the combined effect of these two stressors on the physical function of cancer patients, as we actually want to counteract them simultaneously in order to ultimately improve the survival and quality of life of these patients.

Cancer is a complex disease with various stages and a multitude of available antineoplastic treatments. As a consequence, the patient population can be heterogeneous. In order to avoid high variability between our participants, we decided to limit our inclusion criteria (Table 1) to the most frequent stages encountered at diagnosis in our clinical setup. There are still substantial differences in the treatment between participants; however, what matters in our experimental context is to assess the potential maladaptations of the disease with the treatment that is supposed to be the most optimal to their individual condition. This approach is driven by the end goal of our research projects, which is to determine the best-tailored exercise interventions for cancer patients, and not to assess the net efficacy of one specific treatment.

Finally, the physical activity level will likely differ between patients as well as individually throughout the experiment. As we will be able to control for this variable using the GPAQ score, this will be a great opportunity to provide preliminary insights on the effect of physical activity on our measurements prior to interventional training studies.

## Ethics Statement

The protocol was approved by the French Ethics Committee (Comité de Protection des Personnes Sud-Ouest et Outre Mer III, number ID RCB: 2019-A00848-49). All subjects gave written informed consent in accordance with the Declaration of Helsinki.

## Author Contributions

JM, EH, RS, TP, MD, CB, MB, HC, PC, CF, MK-W, AB, SD, FF, XP, TH, and AP contributed to the study design as well as writing and/or editing the manuscript and approved the final version of the manuscript. All authors contributed to the article and approved the submitted version.

## Conflict of Interest

The authors declare that the research was conducted in the absence of any commercial or financial relationships that could be construed as a potential conflict of interest.
